# Dysregulated T-cell homeostasis and decreased CD30^+^ Treg proliferating in aplastic anemia

**DOI:** 10.1016/j.heliyon.2024.e35775

**Published:** 2024-08-03

**Authors:** Nannan Sun, Mengmeng Zhang, Jingjing Kong, Jin Li, Yong Dong, Xiaoqian Wang, Liyan Fu, Yiwei Zhou, Yaoyao Chen, Yingmei Li, Xianlei Sun, Rongqun Guo

**Affiliations:** a Department of Hematology, The First Affiliated Hospital of Zhengzhou University, Zhengzhou, Henan, China; b Department of Immunology, School of Basic Medical Sciences, Chengdu Medical College, Chengdu, Sichuan, China; c Department of Clinical Laboratory, The First Affiliated Hospital of Zhengzhou University, Zhengzhou, Henan, China; d Department of Laboratory Medicine, The First Clinical College of Henan University of Chinese Medicine, Zhengzhou, Henan, China; e Basic Medical Research Center, Academy of Medical Sciences, Zhengzhou University, Zhengzhou, Henan, China

**Keywords:** Single-cell RNA sequencing, Aplastic anemia, CD30 (TNFRSF8), T cell homeostasis

## Abstract

Aplastic anemia (AA) is an autoimmune hematopoietic disease mediated by autoreactive T cells leading to bone marrow failure. However, the precise role of autoreactive T cells in the development of AA is not fully understood, hindering the advancement of therapeutic and diagnostic strategies. In this study, we conducted a single-cell transcriptome analysis of CD8^+^ T cells, conventional CD4^+^ T (CD4^+^ Tconv) cells, and Treg cells, to elucidate the potential disruption of T cell homeostasis in patients with AA. We identified changes in CD4^+^ Tconv cells, including loss of homeostasis in naïve and memory cells and increased differentiation potential in T helper type 1 (TH1), T helper type 2 (TH2), and T helper type 17 (TH17) cells. Additionally, we identified naïve and memory CD8^+^ T cells that were enforced into an effector state. CD127 is an ideal surface marker for assessing the immune state of CD8^+^ T cells，as identified by flow cytometry. Abnormal expression of TNFSF8 has been observed in AA and other autoimmune diseases. Flow cytometry analysis revealed that TNFRSF8 (CD30), a receptor for TNFSF8, was predominantly present in human Treg cells. Importantly, patients with AA have a decreased CD30^+^ Treg subset. RNA-sequencing analysis revealed, that the CD30^+^ Treg cells are characterized by high proliferation and a remarkable immunosuppressive phenotype. Taken, together, we propose that abnormal TNFSF8/TNFRSF8 signaling is involved in dysfunctional T cell immunity by increasing the destruction of CD30^+^ Treg cells.

## 1 Introduction

AA is characterized by autoreactive T cell-mediated bone marrow (BM) failure with depletion of hematopoietic stem and progenitor cells (HSPCs). However, the reasons for the T cell-mediated depletion of HSPCs are still unclear. TH1, TH2, and TH17 are known to be significantly higher in patients with AA than in healthy individuals [1]. Treg cells from patients with AA have low immunosuppressive abilities [2]. Interestingly, unknown antigens may be involved in the expansion of TH1 cells, thereby mediating the functional impairment of Treg cells by maintaining an inflammatory environment. Lucia Gargiulo et al. reported that increased glycosylphosphatidylinositol (GPI)-specific T cells may be involved in impairing GPI-positive normal hematopoiesis in patients with paroxysmal nocturnal hemoglobinuria (PNH) and AA [3]. Abnormal STAT1 hyper-activation is involved in the progression of AA, suggesting that JAK inhibitors can be used to treat some patients with AA [4]. Several studies have demonstrated the dominant role of autoreactive T-cells [5], although the mechanisms underlying HSPCs destruction are controversial and diverse.

Single-cell RNA sequencing (scRNA-seq) provides unparalleled advantages in exploring the molecular mechanisms of disease pathogenesis [[6], [7], [8], [9]] and immune cell development [10,11]. A study using scRNA-seq analysis identified that selective repression of lineage-committed progenitors, coupled with defects in alternative splicing and polyadenylation, contribute to the dysfunction of HSPCs [12]. Epitopes present on HSPCs can be incorrectly recognized by T cells as antigens associated with persistent viral infections, leading to the T cell-mediated elimination of HSPCs in patients with AA [13]. Virus has been proposed as an initial trigger for AA has been proposed based on the upregulation of human endogenous retroviruses transcripts in HSPCs [14]. Furthermore, cell type-specific ligand-receptor interactions are critical players in T cell-mediated HSPCs destruction. Cytotoxic CD8^+^ T cells induce the destruction of HSPCs via apoptosis and pyroptosis, which is mediated by FASLG, TNFSF12, TNF, and GZMB [15]. Another study has suggested that the somatic *STAT3* mutation *p.Y640F* in CD8^+^ cells is involved in the abnormal autoreactive T cells [16]. Together, these studies on AA using scRNA-seq analysis robustly emphasize that the abnormal elimination of HSPCs by hyperactivated T cells is the core event in AA pathogenesis. Limited by the number of samples and technical means, these datasets can only provide us with some individualized participating factors, rather than general pathogenic factors.

Interestingly, another study involving scRNA-seq analysis of AA BM implied that B cells may be aberrantly involved in the immune dysfunction of myeloid and T/NK cells and accelerate the process of BM failure [17]. Dysfunctional NK cells weaken immune surveillance and cannot suppress the hyperactivation of myeloid dendritic cells (mDCs) interactions with CD8^+^ T cells [18]. Efferocytosis has been identified as a target of AA treatment in a mouse model, highlighting the involvement of myeloid cells in AA pathogenesis [19]. A deficiency owing to insufficient STAT5 phosphorylation in Treg cells has also been observed in patients with AA [20]. The BM mesenchymal stem cells of patients with AA show significantly elevated senescence [21], which may contribute to dysfunctional BM niche. These studies showed that, in addition to T cells, other cell types are also involved in the pathogenesis of AA.

Herein, we applied flow cytometry, scRNA-seq, and bulk RNA-seq to analyze the immune states of different T cell subsets, including CD4^+^ Tconv cells, CD8^+^ T cells, and Treg cells, and identified the characteristic features of naïve/memory state loss in conventional T cells of patients with AA. Abnormal expression of TNFSF8 indicates that dysfunctional TNFSF8/TNFRSF8 signaling is involved in the pathological processes of AA, which also exists in other autoimmune diseases. Furthermore, we identified a novel CD30^+^ (TNFRSF8, the receptor of TNFSF8) Treg subset with a remarkable immunosuppressive phenotype and robust proliferative activity that was decreased in patients with AA.

## 2 Results

### 2.1 scRNA-seq analysis identifies the dysfunctional T-cell homeostasis

**Fig. 1:**
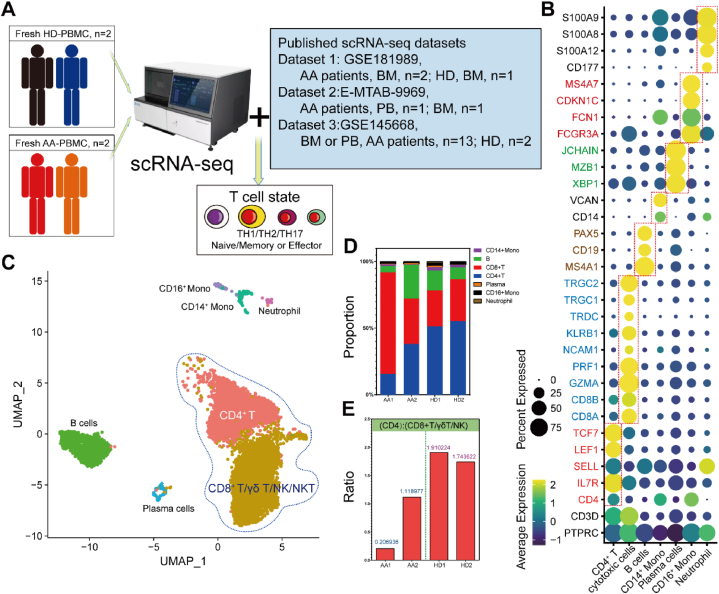
scRNA-seq analysis of PBMCs in patients with AA and HDs. (A). Workflow of experimental strategy. **(B)** Dotplot showing the lineage-specific marker genes. **(C).** UMAP plot generated by pooling the four individual scRNA-seq libraries (patients with AA, n = 2; HD, n = 2). **(D)** Graph depicting the proportion of different immune subpopulations in PBMC samples. **(E)** The ratios of CD4^+^ T cells and cytotoxic cells (CD8^+^ T cells, γδ T cells, and NK cells) across 4 different samples.

To fully capture the immune states of T cells from patients with AA at single-cell resolution, we performed scRNA-seq of fresh peripheral blood mononuclear cells (PBMCs) using the DNelab C4 platform (Fig. 1A and Table S1). Published scRNA-seq datasets (GSE1819889, E-MTAB-9969, and GSE145668) of patients with AA were used to confirm these result (Figs. S1B, S1C, and S1D). We collected fresh PB (<2 h) samples (patients with AA, n = 2; healthy donors [HDs], n = 2) and isolated mononuclear cells (Fig. S1A). Immune cell types were defined based on the expression of lineage-specific genes (CD4^+^ T cells: *CD3D*, *CD4*, *IL7R*, *SELL*, *LEF1*, and *TCF7*; cytotoxic cells [CD8^+^ T/MAIT/γδ-T/NK cells]: *CD3D*, *CD8A*, *CD8B*, *GZMA*, *PRF1*, *NCAM1*, *KLRB1*, *TRDC*, *TRGC1*, and *TRGC2*; B cells: *MS4A1*, *CD19*, and *PAX5*; Plasma cells: *XBP1*, *MZB1*, and *JCHAIN*; CD14^+^ monocytes: *CD14* and *VCAN*; CD16^+^ monocytes: *FCGR3A*, *FCN1*, *CDKN1C*, and *MS4A7;* and neutrophils: *CD177*, *S100A12*, S100A8, and S100A9) (Fig. 1B). Dimensionality reduction and unsupervised clustering of PBMCs yielded seven clusters (Fig. 1C). The percentage of CD8^+^ T cells was markedly increased in patients with AA (Fig. 1D). The ratio of CD4^+^ T cells and cytotoxic cells (CD8^+^ T cells, γδ T cells, and NK cells) was decreased in the patients with AA, which indicated the increased proportion of cytotoxic cells is a notable feature (Fig. 1E). Altogether, AA-related immune cell transcriptomic map were generated to assess the immune dysfunction.

### 2.2 Naïve/memory state loss of CD4^+^ T cells in patients with AA

**Fig. 2:**
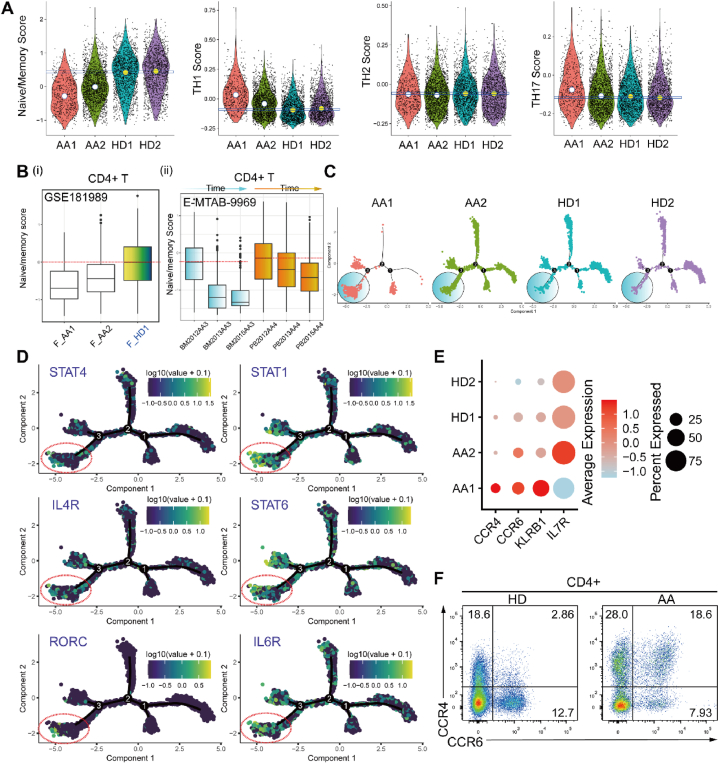
Dysregulated homeostasis of naïve/memory CD4^+^ T cells and increased the differentiation of TH1, TH2, and TH17. (A). The scores of Naïve/memory, TH1, TH2, and TH17 gene sets in BM or PB CD4^+^ T cells of AA patients and HDs. **(B) (i)** Naïve/memory score in BM CD4^+^ T cells of AA patients and HDs (GSE181989 and E-MTAB-9969). The information on patients with AA and HDs can be obtained from Hu Tonglin et al. [17]. **(B) (ii).** Changes of naïve/memory, TH1, TH2, and TH17 scores in PB or BM CD4^+^ T cells of AA patients were monitored during immunosuppressive therapy (IST) using dataset E-MTAB-9969. Patient AA-4, a 52-year-old female, exhibited a somatic *STAT1* mutation P.Y640F in CD8^+^ T cells. Patient AA-3, a 58-year-old female, presented with sAA following two years of macrocytosis and mild thrombocytopenia, and displayed somatic mutations of *KRAS*, *NFATC2*, *PTPN22*, and *TNFAIP3*. The treatment response information can be referenced from Sofie Lundgren et al. [16]. **(C).** The branched trajectory of CD4^+^ T cells was organized by individuals, with each dot representing a single cell. **(D).** Expression maps display log-normalized expression of key functional genes, including *STAT4*, *STAT1*, *IL4R*, *STAT6*, *RORC*, and *IL6R*, in the differentiation branches of naïve CD4^+^ T into TH1/TH2/TH17 cells. Data is represented as log-normalized expressions With yellow indicating high expression and dark blue indicating low expression. **(E).** Dot plots show the expression levels of *CCR4*, *CCR6*, *KLRB1*, and *IL7R* in CD4^+^ T cells of AA patients and HD. **(F).** Representative flow cytometry dot plots illustrate the expression of CCR4 and CCR6 in CD4^+^ T cells from PB samples of AA patients and HDs. (For interpretation of the references to colour in this figure legend, the reader is referred to the Web version of this article.)

To investigate the functional changes in CD4^+^ T cells during the pathogenesis of AA, we extracted CD4^+^ T cells and investigated the T-helper-related gene scores, including TH1 genes (*CCR1*, *CCR5*, *CXCR3*, *IFNGR1*, *IFNGR2*, *IL12RB2*, *IL18R1*, *IL2*7RA, *STAT1*, *STAT4*, *TBX21*, *IFNG*, *IL2*, *TNF*, and *LTA*), TH2 genes (*IL1RL1*, *CRLF2*, *CCR3*, *CCR4*, *CCR8*, *CXCR4*, *IL4R*, *IL1*7RB, *GATA3*, *IRF4*, *STAT5*, *STAT6*, *IL4*, *IL5*, *IL9*, *IL10*, *IL13*, and *IL21*), TH17 genes (*IFNG*, *IL17F*, *IL22*, *CCL20*, *IL23R*, *FGF1*, *FGF12*, *IL17A*, *IL21*, *IL22*, *IL26*, *BATF*, *IRF4*, *RORA*, *RORC*, *STAT3*, *CCR4*, *CCR6*, *IL6R*, *IL1R1*, *TGFBR2*, *IL21R*, and *IL23*), and naïve/memory genes (*CCR7*, *TCF7*, *LEF1*, and *SELL*). The scores of TH1, TH2, and TH17 T cells in patients with AA were higher than those in HDs, whereas the naïve/memory scores were significantly decreased among patients with AA (Fig. 2A and B, Fig. S2A, S2B, and S2C). Although IST treatment can decelerate the trends of TH1, TH2, and TH17 at some stages and in some patients, overall, it did not reverse the loss of the naïve/memory state (Fig. 2B). Subsequently, we predicted the differentiation trajectory of CD4^+^ T cells and found that the CD4^+^ T cells of patients with AA were driven into a highly differentiated state (Fig. 2C). This highly differentiated branch showed the TH1 pattern (such as *STAT4* and *STAT1*), TH2 pattern (such as *IL4R* and *STAT6*), and TH17 pattern (such as *RORC* and *IL6R*) (Fig. 2D and Fig. S2D). IFN-γ and IL-12-mediated STAT1/STAT4 signaling are important for differentiation and maintenance of the TH1 state [22]. The IL4-STAT6-GATA3 axis is the dominant driver of TH2 cell differentiation [23]. IL6R, STAT3, RORC, and BATF are important components of the TH17 regulatory network [24]. This evidence demonstrated a propensity of CD4^+^ T cells to transition away from a naïve/memory state in patients with AA.

Some surface markers, such as CCR4, CCR6, and KLRB1, can help us identify specific TH subsets [25]. We investigated the expression levels of *CCR4* and *CCR6* and found that these genes were upregulated in AA CD4^+^ T cells compared with those of HDs (Fig. 2E and Fig. S2E). Moreover, treatment did not inhibit the upregulation of these genes (Fig. S2F). As described in our previous study, the mRNA level is not always consistent with the protein level at single cell level [8]. Next, we investigated the CCR4, CCR6, and CD161 expression of CD4^+^ T cells among PBMCs by conducting flow cytometry. The frequency of CCR4^+^ cells was significantly higher than that in HDs (Fig. 2F and Fig. S2G). CCR4 is mainly expressed in TH2 cells [26], which can help to identify the proportion of TH2 cells in patients with AA. CCR4 affects T cell retention in different tissues [27] and is required for antigen-specific TH2 cells to efficiently enter inflamed tissues [26]. CCR6 is mainly expressed in TH17 cells [28,29], which can help identify the proportion of TH17-like cells in patients with AA. The proportion of CCR6^+^CD4^+^ T cells was increased in CD4^+^ T cells of patients with AA. Together, the proportion of naïve/memory-like CD4^+^ T cells in patients with AA decreased, and a combination of the expansion of TH1, TH2, and Th17 cells was a distinctive feature of AA.

### 2.3 Naïve/memory state loss of CD8^+^ T cells in AA

**Fig. 3:**
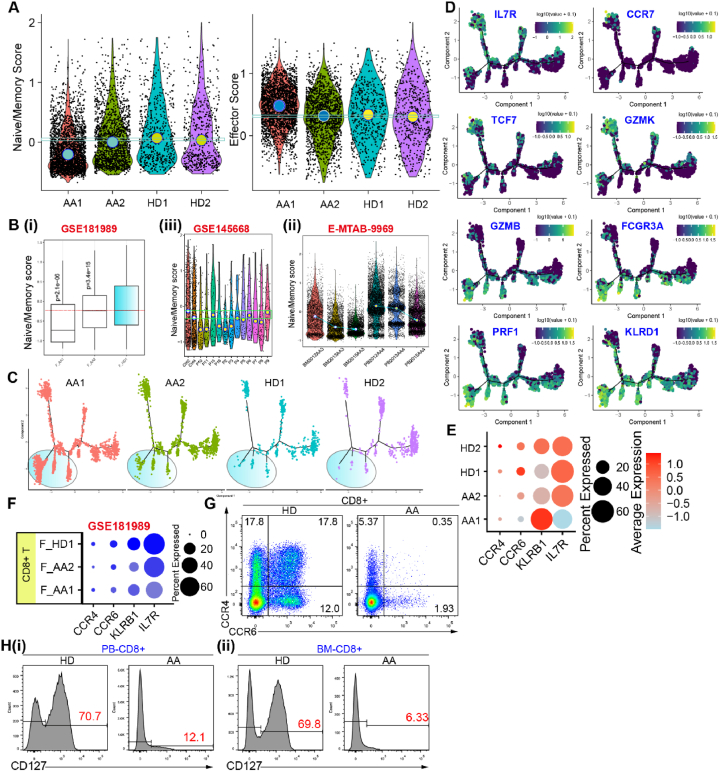
Dysregulated naïve/memory CD8^+^ T cell homeostasis. (A). Naïve/memory score and effector score in PB CD8^+^ T cells of patients with AA and HDs. **(B).** Naïve/memory score in PB or BM CD8^+^ T cells of patients with AA and HDs was analyzed using GSE181989 and GSE145668. Naïve/memory scores in PB or BM CD8^+^ T cells of patients with AA were also tracked during the IST treatment (E-MTAB-9969). **(C).** The branched trajectory of CD8^+^ T cells grouped by individuals. **(D).** Expression maps showing log-normalized expression of marker genes (*IL7R*, *CCR7*, *TCF7*, *GZMK*, *GZMB*, *FCGR3A*, *PRF1*, and *KLRD1*) in the differentiation of Naïve CD8^+^ T into effector cells. Data are shown as log-normalized expressions. **(E).** The dot plot showing the expression levels of *CCR4, CCR6, KLRB1*, and *IL7R* in CD8^+^ T cells from patients with AA and HDs. **(F).** The dot plot showing the expression levels of *CCR4, CCR6, KLRB1*, and *IL7R* in CD8^+^ T cells from patients with AA and HD (GSE181989). **(G).** Representative flow cytometry depicting CD8^+^ cytotoxic cells expressing CCR4 and CCR6 in PB samples of patients with AA and HDs. **(H).** Representative flow cytometry density plots illustrating CD8^+^ T cells expressing CD127 in PB **(i)** and BM **(ii)** samples of AA patients and HDs.

We focused on CD8^+^ T cells and assessed the naïve/memory (geneset: *CCR7*, *TCF7*, *LEF1*, *SELL*, and *IL7R*) score and effector (geneset: *PRF1*, *GZMA*, *GZMB*, *NKG7*, *KLRG1*, *FCGR3A*, *IL2*, *TNF*, and *IFNG*) score of AA CD8^+^ T cells. The results showed that the naïve/memory score significantly decreased in patients with AA compared to HDs (Fig. 3A and B(i), 3B(ii), Figs. S3A and S3B). Treatment did not reverse the trend of the naïve/memory state loss (Fig. 3B(iii)). Interestingly, the effector score did not always increase (Figs. S3A and S3B), suggesting the presence of a pathological state distinct from the naïve/memory and the effector states. We then investigated the differentiation trajectories of CD8^+^ T cells and found specific terminal trajectories in AA samples, as shown in Fig. 3C. These terminal trajectories expressed effector genes (such as *GZMB*, *FCGR3A*, *PRF1*, and *KLRD1*) but not *GZMK* or naïve/memory features (such as *IL7R*, *CCR7*, and *TCF7*) (Fig. 3D and Fig. S3E).

*CCR4*, *CCR6*, *CD127*，and *KLRB1* expression were lower in patients with AA than in HDs (Fig. 3E). CCR4 and CCR6 are mainly expressed in the central and early effector memory CD8^+^ T cells, respectively [30]. KLRB1 is also a potential marker of memory CD8^+^ T cells [31]. IL7R is a memory marker of CD8^+^ T cells [32]. Furthermore, treatment did not increase the expression of *CCR4*, *CCR6*, *KLRB1*, or *IL7R* (Fig. S3E). The results of the surface protein detection assays were consistent with the mRNA expression results (Fig. 3F and G). We found that the frequencies of CCR4^+^CD8^+^, CCR4^+^CCR6^+^CD8^+^, and CCR6^+^CD8^+^ T cells decreased in patients with AA (Fig. 3G and Fig. S3F). Furthermore, in line with the mRNA level, CD8^+^ T cells decreased the expression of CD127 protein, as evidenced by flow cytometric analysis of PB and BM in patients with AA (Fig. 3H and Fig. S3G). The cellular composition of PB T cells varies from that of BM, particularly in terms of the relative abundance of the CCR4^+^CD127^−^CD8^+^ subset and CCR4^−^CD127^+^CD8^+^ subset (Fig. S3H). Together, these results suggest that CD8^+^ T cells of patients with AA decrease the naïve/memory subsets, and that CCR4, CCR6, and CD127 can be used to monitor the naïve/memory state.

### 2.4 Impaired immunosuppressive function of Treg cells and decreased CD30^+^ Treg cells in AA

**Fig. 4:**
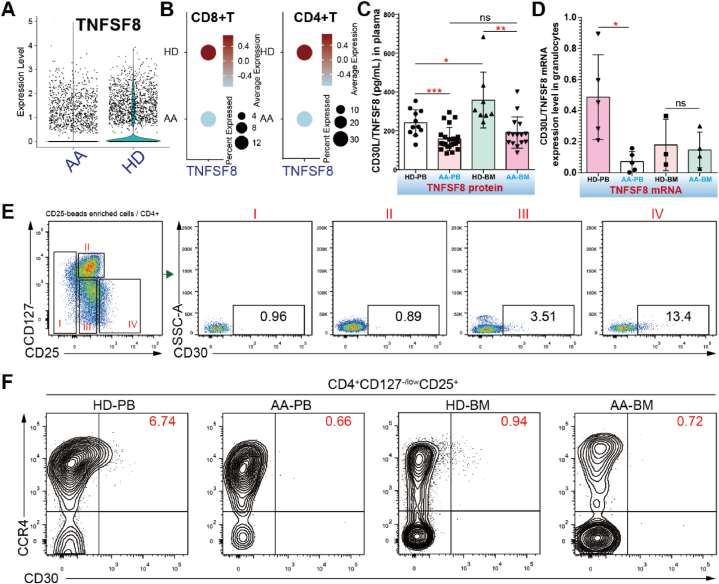
Impairment of Treg suppressive capacity and reduction in the proportion of CD30^+^ Treg cells in AA patients. (A). The violin plots show the expression levels of *TNFSF8* in T cells of patients with AA and HD. **(B).** The dot plots depict the expression levels of *TNFSF8* in PB CD8^+^ T and CD4^+^ T cells from patients with AA and HDs. **(C)**. ELISA was used to compare the serum levels of TNFSF8 in PB (AA, n = 22; HD, n = 11) and BM (AA, n = 15; HD, n = 8) samples from AA patients and HDs. **(D**) The mRNA expression levels of *TNFSF8* in granulocytes from the AABM (n = 4), AAPB (n = 5), HDPB (n = 5), and HDBM (n = 3) groups were assessed using quantitative polymerase chain reaction (qPCR) analysis, with statistical significance indicated as *P < 0.05, **P < 0.01, ***P < 0.001. **(E).** The proportion of CD30^+^ subset within various CD4^+^ T subsets (I: CD25low, II: CD127+CD25^+^, III: CD25^+^CD127-, IV:CD127-CD25high) enriched by CD25-beads from PB of a healthy donor was examined. **(F).** Representative flow cytometry dot plots were used to illustrate the presence of CCR4 and CD30 in CD4^+^CD127-/lowCD25+ Treg cells in PB and BM samples from patients with AA and HDs.

Our findings indicated that the expression of TNFSF8 in CD3^+^ T cells derived from patients with AA was notably reduced compared to that in control individuals (Fig. 4A). Furthermore, both CD4^+^ and CD8^+^ T cells from patients with AA exhibited decreased TNFSF8 expression (Fig. 4B). Subsequently, we examined the expression of *TNFSF8* in other human tissues (reactive normal lymph node [rLN], BM, and spleen) using scRNA-seq and identified CD4^+^ cells as the major source of TNFSF8 (Figs. S4A–C). CD30 ELISA was performed to assess the soluble CD30 protein levels in PB and BM plasma samples of patients with AA and HDs (Fig. 4C). Consistent with our scRNA-seq analysis, PB and BM plasma samples from patients with AA had higher CD30 protein levels than those from HDs. A higher concentration of CD30 protein in PB plasma samples compared with BM samples. We then assessed the expression of TNFSF8 in granulocytes obtained from patients diagnosed with AA and HDs and found a significant downregulation of TNFSF8 mRNA in AA PB-derived granulocytes compared to their corresponding HD counterparts (Fig. 4D).

Interestingly, its receptor, TNFRSF8 (CD30), was enriched in Treg cells (Fig. 4A(ii)), and our flow cytometry results confirmed this at the protein level (Fig. S4D). Surface CD30 is enriched in CD25^+^ cells. We found that Treg cells had a higher proportion of the CD30 subset than conventional CD4^+^ T cells (I/II) (Fig. 4E). More importantly, CD25^high^ Treg cells showed higher expression levels of CD30 than CD25^+^ Treg cells. The CD25^high^ Treg subset possessed higher suppressive potency and dependence on IL-2 for TCR-induced proliferation than the CD25^+^ Treg subset [33]. The expression levels of immunosuppressive genes in the CD30^+^ Treg subset were higher than those in their counterparts (Fig. S4A(iii)). These results imply that CD30^+^ Treg subset might be involved in the Treg reduction in patients with AA mediated by dysfunctional conventional T cells (Fig. S4E). To confirm our hypothesis, we compared the CD30 expression levels of Treg cells from patients with AA and those from HDs (Fig. 4F). The proportion of CD30^+^ Treg cells was significantly decreased in PB and BM sample-derived Treg cells from patients with AA (Fig. S4F). Furthermore, CD30^+^ Treg cells were mainly localized in the CCR4^+^ Treg population. Our data consistently demonstrated that the immunosuppressive function and proliferative potency of Treg cells were impaired in patients with AA, and that CD30^+^ Treg cells could be regarded as a reliable index to assess Treg function and proliferative ability.

### 2.5 The expression pattern of CD30 differs between mouse and human immune cells

**Fig. 5:**
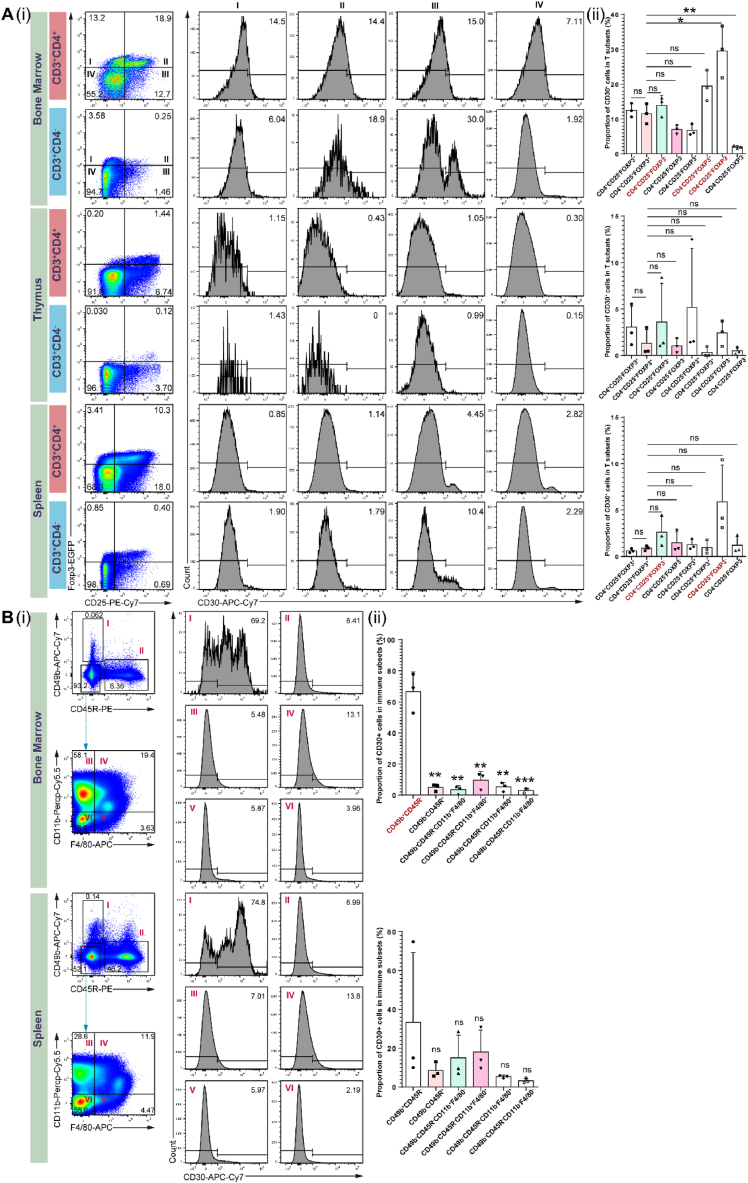
The expression of CD30 in various mouse immune populations. (A). Flow cytometry gating strategy for identifying CD30^+^ cells in T cells from mouse BM, thymus, and spleen **(i)**. Quantification of the percentage of CD30^+^ cells within indicated T cell populations from BM, thymus, and spleen **(ii). (B).** Flow cytometry gating strategy for identifying CD30^+^ cells in NK/NKT cells, B cells, and myeloid cells from mouse BM, thymus, and spleen **(i)**. Percentage of CD30^+^ cells in indicated non-T cell populations in BM, thymus, and spleen **(ii)**.

To further investigate the relationship between CD30 and Treg cells, we planned to establish a mouse model of AA. First, we analyzed CD30 expression in different BM, thymus, and spleen immune cell types in FOXP3-EGFP C57BL/6 mice (Fig. 5A(i)). Unfortunately, the CD30 expression patterns in mouse immune cell types were inconsistent with those in human T cells. CD30 was not enriched in mouse Treg cells (CD3^+^CD4^+^CD25^+^FOXP3^+^), but was enriched in CD25^+^ T cells (CD3^+^CD4^−^CD25^+^FOXP3^+/−^) (Fig. 5A(ii)), indicating AA mouse model may not be suitable for mimicking the relationship between CD30 and Treg cells in humans. Interestingly, mouse NK/NKT cells (CD49b^+^CD45R^−^) expressed surface CD30 at high levels in the BM (Fig. 5B). Therefore, we did not use an AA mouse model to assess the relationship between CD30 molecules and Treg cells.

### 2.6 TNFSF8 expression of CD4^+^ T cells is downregulated in several autoimmune diseases

**Fig. 6:**
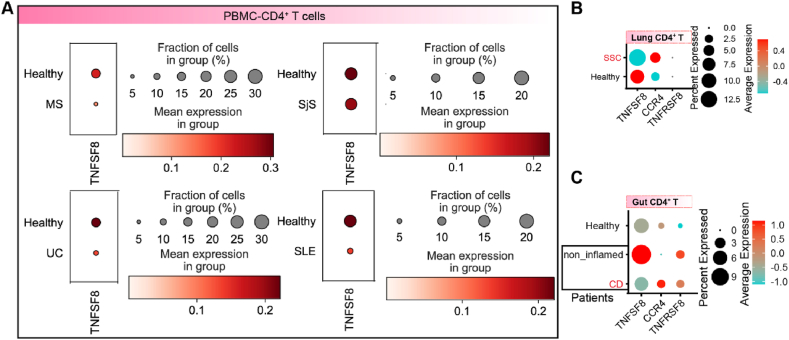
Abnormal expression of TNFSF8 was observed in CD4^+^ T cells of several autoimmune diseases (MS, SjS, UC, SLE, SSc-ILD, and CD). (A). The dot plots were used to illustrate the average and proportion of TNFSF8 expression in PBMC CD4^+^ T cells of MS, SjS, UC, and SLE patients. The expression levels of TNFSF8 in CD4^+^ T cells were compared between patients with SSc-ILD **(B)**, patients with CD **(C)**, and their respective control samples.

To identify the expression patterns of *TNFSF8* in other autoimmune diseases, we explored other datasets based on Interactive Analysis and Atlas for Autoimmune disease (http://galaxy.ustc.edu.cn/IAAA) [34]. As illustrated in Fig. 6A, PBMC-derived CD4^+^ T cells from patients with multiple sclerosis (MS), Sjögren's syndrome (SjS), ulcerative colitis (UC), and systemic lupus erythematosus (SLE) showed lower TNFSF8 expression levels than those from the control groups (Fig. 6A). We also analyzed the scRNA-seq datasets of systemic sclerosis-associated interstitial lung disease (SSc-ILD) patient-derived lung tissue and Crohn's disease (CD) patient-derived gut tissue (Figs. S5A and S5C). CD4^+^ T cells are the major source of *TNFSF8* and Treg cells express *TNFRSF8* transcripts at high levels (Figs. S5B and S5D). Compared to control samples, patient-derived lung CD4^+^ T cells for SSc-ILD and CD showed lower *TNFSF8* expression (Fig. 6B and C). These results demonstrate that dysfunctional TNFSF8-TNFRSF8 signal might be involved in T cell dysfunction. In addition, the CD4^+^TNFSF8^high^ subset expressed *NR3C1* at higher levels than the CD4^+^TNFSF8^+^ subset Figs. S5E and S5F).

### 2.7 CD30^+^ Treg subset is characterized as a novel proliferating Treg population with robust immunosuppressive activity

**Fig. 7:**
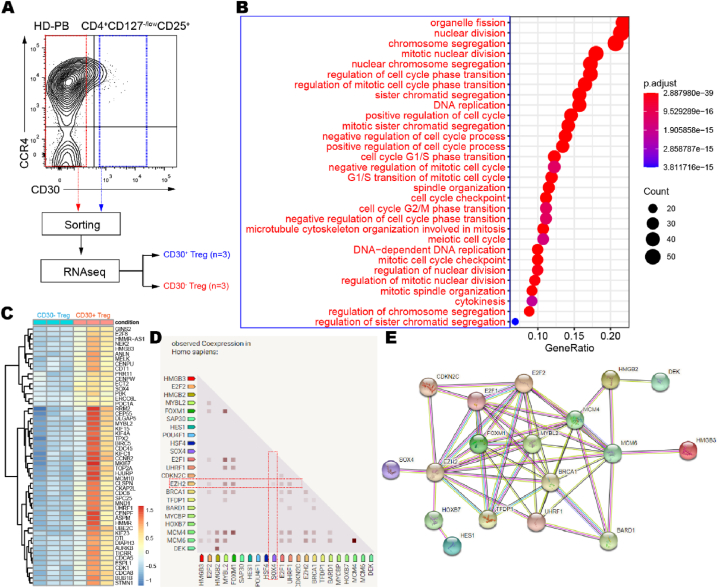
RNA-seq analysis demonstrates the potent immunosuppressive characteristics of proliferating CD30^+^ Treg cells. (A). The sorting strategy employed for bulk RNA-seq of CD30^+^ Treg cells and CD30^−^ Treg cells. **(B).** Representative GO terms enriched in upregulated DEGs of CD30^+^ Treg cells. **(C).** Heatmap showing the cell cycle and proliferation signatures between CD30^−^ Treg cells and CD30^+^ Treg cells. **(D).** Heatmap showing the coexpression scores of upregulated differential transcription factors of CD30^+^ Treg cells obtained from the STRING database (https://cn.string-db.org/cgi/input?sessionId=bhtjZNyicyBa&input_page_show_search=on). **(E).** Coexpression relationships between upregulated differential transcription factors in CD30^+^ Treg cells.

To explore the difference between CD30^+^ Treg and CD30^−^ Treg subsets, we sorted both CD30^+^ Treg cells and CD30^−^ Treg cells from HD PBMCs and performed bulk RNA-seq analysis (Fig. 7A). Gene ontology (GO) analysis showed that almost all differential gene sets were associated with cell proliferation and cell cycle (Fig. 7B). CD30^+^ Treg cells expressed high levels of cell-cycle/proliferating-related genes (*GINS2*, *E2F8*, *HMMR*-*AS1*, *NEK2*, *HMGB3*, *ANLN*, *MYBL2*, *CCNB2*, *MIK67*, *TOP2A*, and *CDC6*) at high levels (Fig. 7C). We then analyzed the differential transcription factors (TFs) and found that the CD30^+^ Treg subset expressed several important factors, including *MCM4*, *HMGB3*, UHRF1, *HMGB2*, *TFDP1*, *E2F1*, *BRCA1*, *MCM6*, *FOXM1*, *MYBL2*, *CDKN2C*, *E2F2*, *BARD1*, *DEK*, *SAP30*, *POU4F1*, *MYCBP* and *HSF4* (Fig. S6A). GO analysis showed that these TFs were involved in the cell cycle/proliferation (Fig. S6B). Co-expression analysis showed the co-expression of EZH2 and SOX4 (Fig. 7D), which is consistent with a previous report [35]. Protein-protein interaction (PPI) analysis of these differential TFs showed that the core TFs were cell cycle/proliferation-related genes (Fig. 7E). EZH2 increases the stability of FOXP3 and inhibits the production of pro-inflammatory cytokines by Treg cells [36]. In rheumatoid arthritis (RA), IL17 in synovial fluid may be involved in the downregulation of EZH2 in CD4^+^ T cells [37], implying that enhanced TH17 differentiation may impair the function of the CD30^+^ Treg subset. Furthermore, SOX4 is a critical regulatory factor for CD39 expression, which is consistent with our bulk RNA-seq data (Fig. S6C). Our bulk RNA-seq data also showed that CD30^+^ Treg cells had a high expression of immunosuppressive genes, such as *CTLA4*, *ENTPD1*, *LGALS1*, *NT5E*, *LAG3*, *CD200R*, and *CD274*. Furthermore, CD30^+^ Tregs exhibit high FAS expression levels. Altogether, we identified a novel functional CD30^+^ Treg subset that can be used to assess the immunosuppressive ability and proliferative state of Treg cells.

## 3 Discussion

Our findings clearly illustrated the role of scRNA-seq in the pathogenetic analysis of AA and identified the conversion from a naïve/memory state to an effector or dysfunctional state in conventional T cells. High percentages of IFN-γ^+^ and perforin^+^CD5^+^ T cells predicted poor response to immunosuppressive therapy in AA [38], which also implied that the naïve/memory state loss of T cells was related to the AA progression and severity. Other cell types, such as dendritic cells (DCs), have been implicated in abnormal immune responses. mDCs can activate effector T cells mediated by cofilin-1 in patients with AA [39]. Phagocytosis of mDCs in patients with severe AA (SAA) was positively correlated with the levels of IL-2 and IL-4, suggesting that not only TH1 but also TH2 are related to abnormal mDC function [40]. The TH1/TH2 ratio, an immunological and clinical indicator of AA, seems unreliable. Our results suggest that the naïve/memory state index of T cells could eliminate the confusion of personalized T cell subsets owing to the complexity and diversity of the patient's physical condition. Based on the scRNA-seq analysis, we assessed the potentials and trends of T helper lineages under AA conditions in higher dimensions. A simple combination of several antibody surface markers has some limitations in defining specific T helper subsets [41]. Our results showed that CCR4, CCR6, and KLRB1 could be used as negative markers of naïve/memory states in CD4^+^ T cells. In contrast, the expression of CCR4 and CCR6 was downregulated in the CD8^+^ cells of patients with AA. Therefore, we hypothesized that *CCR6* mRNA upregulation in bulk PBMCs and bone marrow mononuclear cells (BMMCs) is mainly caused by CCR6^+^CD4^+^ effector T cells. CCR4^+^CD8^+^ T cells are immature memory CD8^+^ T cells that do not express perforin or granzyme A/B [42]. More importantly, CD127 is an ideal indicator of the degree of naïve/memory state loss in CD8^+^ T cells.

Importantly, we found that TNFSF8 expression was decreased in the T cells of patients with AA, which can also be found in other autoimmune diseases, such as CD, SLE, and UC. Downregulation of TNFSF8 in T cells occurs in multiple human autoimmune diseases, indicating the existence of a common regulator of the TNFSF8-TNFRSF8 axis. TNFRSF8/CD30 is the receptor for TNFSF8/CD153, and is related to proliferative or anti-apoptotic signaling [43]. TNFSF8 is a product of activated myeloid cells and positively correlates with the severity of UC [44]. TNFSF8 is an important risk factor of inflammatory bowel disease (IBD) in East Asians [45]. Numerous studies have reported that TNFSF8 is involved in autoimmune and inflammatory diseases [[46], [47], [48], [49], [50]]. The interaction between TNFSF8 and TNFRSF8-triggered signaling in CD4^+^ T cells is involved in the pathogenesis of Sjögren's Syndrome [51]. Although the TNFSF8 mRNA level in T cells is not consistent with the TNFSF8 protein levels in the plasma, the elevated TNFSF8 protein level in the plasma strongly implicates abnormal TNFSF8/TNFRSF8-mediated signaling involved in dysfunctional immune regulation. Our results also indicated that TNFSF8-triggered signaling is involved in the pathogenesis of autoimmune diseases. Unlike previous studies, our study further found that the CD25^high^ Treg subset expressed TNFRSF8 at a high level, but not other T subsets (conventional T cells and the CD25^+^ Treg subset), which suggests thatTNFSF8 may involve in the proliferation or survival of Treg cells. A mouse model of immune-mediated glomerulonephritis showed that the blockade of CD30 is a potential therapeutic strategy that may lead to the inhibition of TH17 cell proliferation and migration [52]. Further studies are required to clarify the relationship between conventional T-cell-derived TNFSF8 and the CD30^+^ Treg subset. However, there is a huge difference in CD30 gene expression patterns between mice and humans, which limits further exploration of the relationship between CD30 molecules and Treg cells using the AA mouse model. This prompted us to further consider whether the functional information of TNFSF8 based on a mouse model is applicable to human autoimmune diseases.

A previous study reported that the functional molecule CTLA-4 in Treg cells was downregulated in SAA [53], consistent with our scRNA-seq analysis of Treg cells. Furthermore, CTLA4 was expressed at a higher level in the CD30^+^ Treg subset compared with the CD30^−^ Treg subset, and the loss of the CD30^+^ Treg subset resulted in CTLA4 downregulation. CCR4 can be used as a memory/activated marker of Treg cells, and CCR4^+^ Treg cells have a higher CD95 (FAS) expression [54]. In the present study, the CD30^+^ Treg subset was mainly a subset of CCR4^+^ Treg cells and expressed CD95 at a high level. Based on the upregulated genes, the CD30^+^ Treg subset can be regarded as proliferating or expanding Treg cells with high suppressive activity. Irf4 regulates Tnfrsf8 expression, thereby affecting effector Treg cell differentiation [55].

Interestingly, vitamin D3 can increase the expression of TNFRSF8 and other immunosuppressive functional genes, such as *CTLA4*, *IL10*, *IL2RA*, and *IRF4*, also supporting the idea that TNFRSF8 is related to effector Treg cells. Another study has identified TNFRSF8 as a hallmark of activated Treg cells [56]. Although these studies provided information on the relationship between TNFRSF8 and effector or activated Treg cells, CD30^+^ Treg cells must be defined. Our study confirmed that the CD30^+^ Treg subset is a proliferating Treg subset with a remarkable immunosuppressive phenotype.

## 4 Conclusions

Our current study reports an imbalance between naïve/memory conventional T cells and effector T cells in patients with AA. These findings also provide new surface marker combinations (CCR4, CCR6, and CD127) for assessing the functional state of T cells in patients with AA. Dysfunctional TNFSF8/TNFRSF8 signaling exists in AA and other autoimmune diseases. Furthermore, we found a decrease in CD30^+^ Treg cells in patients with AA and defined the CD30^+^ Treg subset as a proliferating Treg subset with a robust immunosuppressive gene expression pattern, which also provides an indicator for assessing Treg function in patients with AA. These results indicate that communication between conventional CD4^+^ T cells and Treg cells mediated by TNFSF8/TNFRSF8 is important for maintaining T-cell homeostasis to avoid autoimmune diseases.

## 5 Materials and methods

### 5.1 Flow cytometry analysis

Venous and BM samples from HDs and patients with AA were collected in EDTA anticoagulant tubes. This study was approved by the Research and Clinical Trial Ethics Committee of the First Affiliated Hospital of Zhengzhou University (KYKS2016-21). PBMCs and BMMCs were isolated within 24 h by isopycnic centrifugation using Ficoll-Paque PLUS (No. 17-1440-03, GE Healthcare). The nonspecific binding of the immunoglobulin to the Fc receptors of PBMCs and BMMCs was blocked by FcR Blocking Reagent (No. 130–0590901, Miltenyi Biotec), and stained with the following antibodies: FITC anti-human CD8a antibody (RPA-T8, No. 301050, BioLegend), APC-Cy7 anti-human CD4 antibody (A161A1, No. 357415, BioLegend), PE anti-human CCR4 (CD194) antibody (L291H4, No. 359412, BioLegend), APC anti-human CCR6 (CD196) antibody (G034E3, No. 353416, BioLegend), Biotin anti-human CD161 antibody (HP-3G10, No. 339932, BioLegend), APC anti-human CD30 antibody (BY88, No. 333910, BioLegend), Percp-Cy5.5 anti-human CD25 antibody (BC96, No. 302626, BioLegend), and PE-Cy7 anti-human CD127 antibody (A7R34, No. 25-1271-82, eBioscience), combined with APC-Cy7 Streptavidin (No. 405208, BioLegend), and PE/Cyanine7 Streptavidin (No. 405206, BioLegend). In some experiments, CD25^+^ cells were enriched with biotin-conjuaged anti-human CD25 antibody (BC96, No. 302624, BioLegend) and anti-biotin microbeads (No. 130-090-485, Miltenyi Biotec). The cells were resuspended in 400 μl of 4′,6-diamidino-2-phenylindole (DAPI) Staining Solution (0.1 μg/mL) (No. C0060, Solarbio) and analyzed using an Aria II cytometer (BD Biosciences). Flow cytometry data were analyzed using FlowJo V10 (Three Star, Ashland OR). Percentage data were presented as mean ± SD using GraphPad Prism 6.

### 5.2 Mouse maintenance and sample preparation

*Foxp3*-*EGFP* (C57BL/6J background) mice were kindly donated by the laboratory of Xuyu Zhou [57] and maintained in an SPF animal facility. Mouse BM, spleen, and thymus were isolated from 8-week-old *Foxp3*-*EGFP* mice, which were made in single-cell suspension using mechanical methods with filtering through 70-μm cell strainers. Ammonium–chloride–potassium (ACK) lysing buffer was used to lyse mouse red blood cells before antibody incubation. The non-specific binding of the immunoglobulin to the Fc receptors of mouse immune cells was blocked by FcR Blocking Reagent (No. 130-092-575, Miltenyi Biotec), and stained with the following antibodies: APC anti-mouse CD3 antibody (17A2, No. 100236, BioLegend), Percp-Cy5.5 anti-mouse CD4 antibody (GK1.5, No. 100434, BioLegend), PE-Cy7 anti-mouse CD25 antibody (PC61.5, No. 25-0251-82, BioLegend), biotin anti-mouse CD30 antibody (mCD30.1, No. 102303, BioLegend), APC-Cy7 anti-mouse CD49b antibody (DX5, No. 108920, BioLegend), and PE anti-mouse CD45R antibody (REA755, No. 130-110-846, Miltenyi Biotec), combined with APC-Cy7 Streptavidin (No. 405208, BioLegend), and PE/Cyanine7 Streptavidin (No. 405206, BioLegend). The cells were resuspended in 400 μl of DAPI Staining Solution (0.1 μg/mL) (No. C0060, Solarbio), and analyzed using an Aria II cytometer (BD Biosciences). Flow cytometry data were analyzed using FlowJo V10 (Three Star). Percentage data were presented as mean ± SD using GraphPad Prism 6.

### 5.3 Determination of CD30L protein

Platelet was removed from the plasma samples, and these platelet-free plasma samples were stored at −80 °C for long-term storage. CD30L levels in plasma samples of PB and BM from patients with AA and HDs (HD-PB, n = 11; HD-BM, n = 8; AA-PB, n = 22; AA-BM, n = 15) were analyzed using a human CD30L (Cluster of Differentiation 30 ligand) ELISA Kit (No. EH0120, FineTest) according to the manufacturer's protocol.

### 5.4 Quantification of CD30L mRNA expression in human granulocytes

Granulocytes and erythrocytes were separated using Ficoll-Paque PLUS, followed by elimination of erythrocytes using Red Blood Cell (RBC) lysis buffer (No. R1010, Solarbio). The samples were transferred into TRIzol (No. 15596026, Invitrogen) and subsequently stored at −80 °C. Subsequently, cDNA synthesis was performed using the HiScript II cDNA Reverse Transcriptase Kit (No. R223, Vazyme) with total RNA as the starting material. Gene expression analysis was conducted using a Quant-Studio 3 machine and SYBR reagent (No. 11202, Yeasen). The following primers were used: *GAPDH*, forward (*ATCAATGGAAATCCCATCACCA*) and reverse (*GACTCCACGACGTACTCAGCG*); *TNFSF8*, forward (*CACGAGCCGCAGCTATTTCTA*) and reverse (*CTCTGAACGACCAACACCATAA*).

### 5.5 Single-cell RNA library construction and sequencing

Venous blood samples from HDs and patients with AA were collected in EDTA anticoagulant tubes. PBMCs were isolated using Ficoll. The DNBelab C Series High-throughput Single-Cell RNA Library (MGI, No. 940-000047-00) was ussed for scRNA-seq library preparation, and the single-cell suspensions were converted to barcoded scRNA-seq libraries as described in the manufacturer's protocol. cDNA was sheared to short fragments of 250–400 bp and indexed sequencing libraries were produced according to the manufacturer's protocol. Qualification was performed using a Qubit ssDNA Assay Kit (No. Q10212, Thermo) and Agilent Bioanalyzer 2100 (Agilent). The libraries were sequenced using the DNBSEQ-T7 platform with pair-end sequencing.

### 5.6 scRNA-seq data processing

All sequencing data were processed using the DNBelab C Series Single-Cell RNA Analysis Software. Briefly, samples were performed with default parameters: sample de-multiplexing, barcode processing, and single-cell 3′ unique molecular identifier (UMI) counting. The processed reads were aligned to the human genome at version 38 using STAR (version 2.5.1b). Valid cells were identified based on the UMI number distribution of each cell using the “barcodeRanks(.)” function of the DropletUtils tool, and PISA was used to calculate the gene expression of cells and create a single cell matrix for each sample.

### 5.7 scRNA-seq datasets and quality control

Cells from our datasets were filtered with defined gene expression numbers (200-10,000) per cell, and the mtDNA% per cell was below 10. Published scRNA-seq datasets of HDs and patients with AA were acquired from the GEO (GSE181989) [17], EMBL-EBI (E-MTAB-9969) [16], and GSE145668 databases [12]. Cells from GSE181989 dataset were filtered with defined gene expression numbers (200-10,000) per cell, and the mtDNA% per cell was below 12.5. Cells from the E-MTAB-9969 dataset were filtered with defined gene expression numbers (200-10,000) per cell, and the mtDNA% per cell was below 20. Cells from the GSE145668 dataset were filtered with a defined gene expression number (200-10,000) per cell, and the mtDNA% per cell was below 15. The scRNA-seq datasets (SSc-ILD lungs, GSE128169; terminal ileum of childhood-onset Crohn'‘s disease, https://cellgeni.cog.sanger.ac.uk/gutcellatlas/pediatric_RAWCOUNTS_cellxgene.h5ad) of patients with systemic sclerosis-associated interstitial lung disease (SSc-ILD) or Crohn's disease (CD) were processed as previously described [9]. Spleen cells from GSE159929 dataset were filtered with a defined gene expression number (200-10,000) per cell, and the mtDNA% per cell was below 15. The scRNA-seq datasets of reactive non-malignant lymph node (rLN) was downloaded from the website https://www.zmbh.uni-heidelberg.de/Anders/scLN-index.html and then filtered with a defined gene expression number (200-10,000) per cell, and the mtDNA% per cell was below 5. Normal human BM cells from the GSE116256 dataset were filtered with a defined gene expression number (200-10,000) per cell, and the mtDNA% per cell was below 10.

### 5.8 Data processing

Datasets were analyzed in the Seurat V4 package [58], and the “NormalizeData” function was used to normalize the single-cell matrix. We processed the datasets using the “FindVariableFeatures,” “ScaleData,” and “RunPCA” functions. Clusters were calculated using the “FlindClusters” function with a resolution of 0.5 was used for calculating the clusters. These clusters were visualized using a uniform manifold approximation and projection (UMAP) dimensional reduction method. The lineage-specific markers are shown in Fig. 1B. Other markers for the published scRNA-seq datasets are listed below. T/NK cells were identified by high expression of *CD3D*, *CD3E, CD3G*, *KLRD1*, *NKG7*, and *CST7*. HSPCs were identified by feature transcripts, including *CD34*, *HOXA9*, *HOXA10*, and *GATA2*, while other subtypes could be identified by lineage-specific genesets, **(a)** erythroid lineages: *GATA1* and *HBB*; **(b)** B cells: CD19 and MS4A1; **(c)** plasma cells: *XBP1* and *MZB1*; **(d)** monocytes: *FCGR3A*, *CD14*, *CSF1R*, and *CSF3R*; **(e)** DCs: *CD1C*, *HLA*-*DPB1*, *HLA*-*DPA1*, *HLA*-*DQA1*, and *ITGAX*; (f) Plasmacytoid DC （pDCs）: *IL3RA*, *JCHAIN*, *IRF7*, and *CLEC4C*. The CD4^+^ T cells, CD8^+^ T cells, NK cells, Treg cells, and γδ-T cells were divided by several genesets (CD4^+^ T: *IL7R*, *CD4*, *CCR7*, *TCF7*, and *LEF1*; CD8^+^ T: *CD8A*, *CD8B*, *GZMK*, *GNLY*, *GZMB*, and *PRF1*; NK: *NCAM1*, *FCGR3A*, *KLRF1*, *KLRC1*, *NKG7*, *CST7*, and *KLRD1*; Treg: *FOXP3*, *IL2RA*, and *CTLA4*; γδ-T: *TRDC*, *TRGC1*, and *TRGC2*). We used the “AddModuleScore” function for calculating module scores based on the expression values of signature gene sets (CD4^+^ T cells: “TH1” module, “TH2” module, “TH17” module, and “Naïve/memory” module; CD8^+^ T cells: “Naïve/memory” module, “Effector” module, and “Exhaustion” module). These gene set scores are presented as violin or box plots.

### 5.9 Bulk RNA-seq analysis of Treg cells

CD30^+^ and CD30^−^ Treg cells were sorted using an Aria II cytometer, and 10,000–100,000 cells per sample were sorted from the PBMCs of HDs. These samples were transferred into TRIzol (No. 15596026, Invitrogen) and stored at −80 °C. Genomic DNA was removed from the RNA solution using DNaseI treatment. RNA quality was assessed by examining A260/A280 with NanoDrop One/One^c^ Microvolume UV–Vis Spectrophotometers (Thermo). RNA was quantified using a Qubit RNA Broad Range Assay Kit (No. Q10210, Life Technologies). Total RNA was used to prepare a stranded RNA sequencing library using the KC-Digital Stranded mRNA Library Prep Kit for Illumina (No. DR08502, Wuhan Seqhealth), and 200-500bp cDNA was enriched for sequencing on a NovaSeq 6000 sequencer (Illumina) using the PE150 model. Raw sequencing data were processed using Trimmomatic, and de-duplicated consensus sequences were mapped to GRCH38 using STAR software. DESeq2 was used to normalize and identify differentially expression genes (DEGs) (*P*-value <0.05, foldchange> 1.5) in the samples [59]. GO enrichment analysis of the DEGs was performed using ClusterProfiler program [60]. Gene co-expression and gene regulation networks of the differentially expressed transcription factors were analyzed using the STRING website (string-db.org) [61].

## Funding

This work was supported by 10.13039/501100001809National Natural Science Foundation of China (No. 82100240, RQ. Guo; U1804192, YM. Li), Youth Fund of the 10.13039/501100016305First Affiliated Hospital of Zhengzhou University (2017) (71804, NN. Sun), the China Postdoctoral Science Foundation (2021M692929, RQ. Guo), the 10.13039/501100013066Key scientific research projects of colleges and universities in Henan Province (22A320016, RQ. Guo), the Postdoctoral Research Start-up Funding of Henan Province (202001006, RQ. Guo), Joint Co-construction Project of Henan Medical Science and Technology Research Plan (LHGJ20200280, RQ. Guo), Provincial and Ministry Joint Co-construction Project of Henan Medical Science and Technology Research Plan (SBGJ202103045, RQ. Guo), Postdoctoral Research Start-up Funding of the 10.13039/501100016305First Affiliated Hospital of Zhengzhou University (RQ. Guo), Key Research and Development and Promotion Project of Henan province (212102310755, RQ. Guo), and Science Fund Program of Henan for Distinguished Young Scholars (232300421049, RQ. Guo).

## Ethics approval

The studies involving human participants were reviewed and approved by the Research and Clinical Trial Ethics Committee of the First Affiliated Hospital of ZZU (2022-KY-0725, 2023-KY-0813, and 2023-KY-0830). The patients/participants provided their written informed consent to participate in this study. The animal protocols for the experiments described in this paper were approved by the Ethical Committee of Zhengzhou University (ZZU-LAC20210604[06]). The study was conducted in accordance with the local legislation and institutional requirements.

## Data availability statement

All relevant data are available from the authors upon reasonable request. The published datasets presented in this study can be found in online repositories. The names of the repository/repositories and accession number(s) can be found in the article. scRNA-seq data sets are available in Figshare (DOI: 10.6084m9.figshare.26044282), which can be downloaded from this website https://figshare.com/s/a6106ec332ad8189da3d. And raw sequence data are available in the National Genomics Data Center (NGDC), Genome Sequence Archive (GSA) database under the accession of HRA007760. Data set of bulk RNA-seq of CD30^+^ and CD30^−^ Treg cells was available in Figshare (https://doi.org/10.6084/m9.figshare.26090650), which can be downloaded from this website https://figshare.com/s/e18f3613ba06cdff3fcc.

## CRediT authorship contribution statement

**Nannan Sun:** Writing – review & editing, Writing – original draft, Validation, Methodology, Funding acquisition, Formal analysis, Data curation. **Mengmeng Zhang:** Writing – original draft, Methodology, Formal analysis, Data curation. **Jingjing Kong:** Validation, Software, Resources, Methodology, Investigation, Data curation. **Jin Li:** Resources, Investigation, Formal analysis, Data curation. **Yong Dong:** Supervision, Software, Methodology, Investigation. **Xiaoqian Wang:** Supervision, Software, Resources, Investigation, Data curation. **Liyan Fu:** Validation, Supervision, Software, Investigation. **Yiwei Zhou:** Supervision, Software, Investigation, Data curation. **Yaoyao Chen:** Supervision, Software, Investigation, Data curation. **Yingmei Li:** Validation, Supervision, Resources, Investigation, Data curation. **Xianlei Sun:** Writing – review & editing, Methodology, Data curation, Conceptualization. **Rongqun Guo:** Writing – review & editing, Funding acquisition, Conceptualization.

## Declaration of competing interest

The authors declare that they have no known competing financial interests or personal relationships that could have appeared to influence the work reported in this paper.
