# Supplementary material for: Dysregulated T-cell homeostasis and decreased CD30+ Treg proliferating in aplastic anemia
Source: Heliyon. 2024 Aug 3;10(15):e35775. doi: 10.1016/j.heliyon.2024.e35775 (PMC11337026; doi:10.1016/j.heliyon.2024.e35775)
Supplement: Multimedia component 1 [file mmc1.docx]

**Supplementary materials**

**
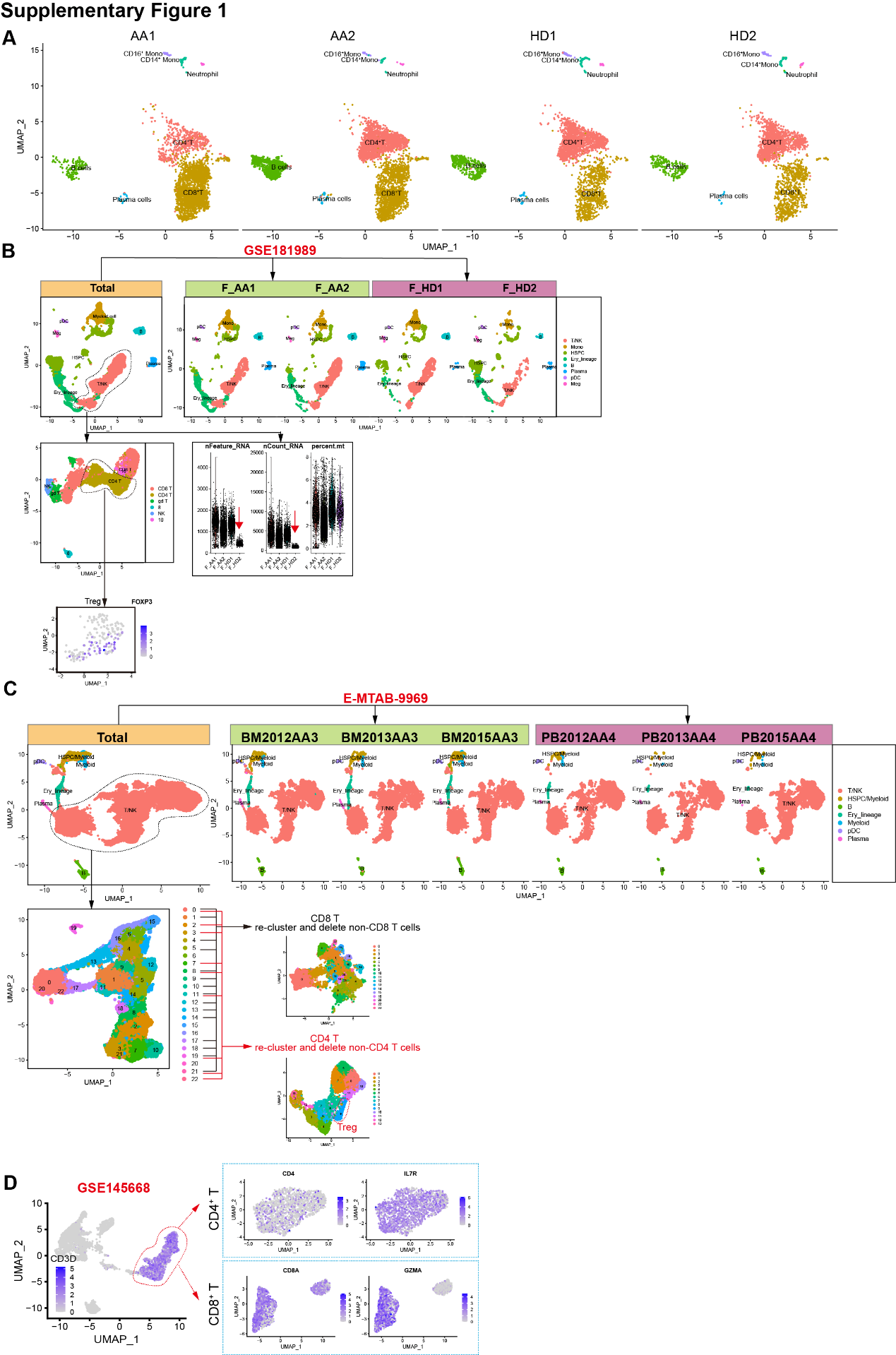
**

**Fig. S1. The single-cell atlas of PBMC and BMMC from patients with AA and HDs. (A).** UMAP plots were generated for each sample. **(B).** Study overview of T subset analysis using the GSE181989 dataset. The dataset from patient F_AA2 exhibited abnormal values of nFeature_RNA and nCount_RNA, leading to exclusion of this data from further analysis (red arrow). **(C).** Study overview of T subset analysis using E-MTAB-9969 dataset. **(D)**. Study overview of T subset analysis using GSE145668.


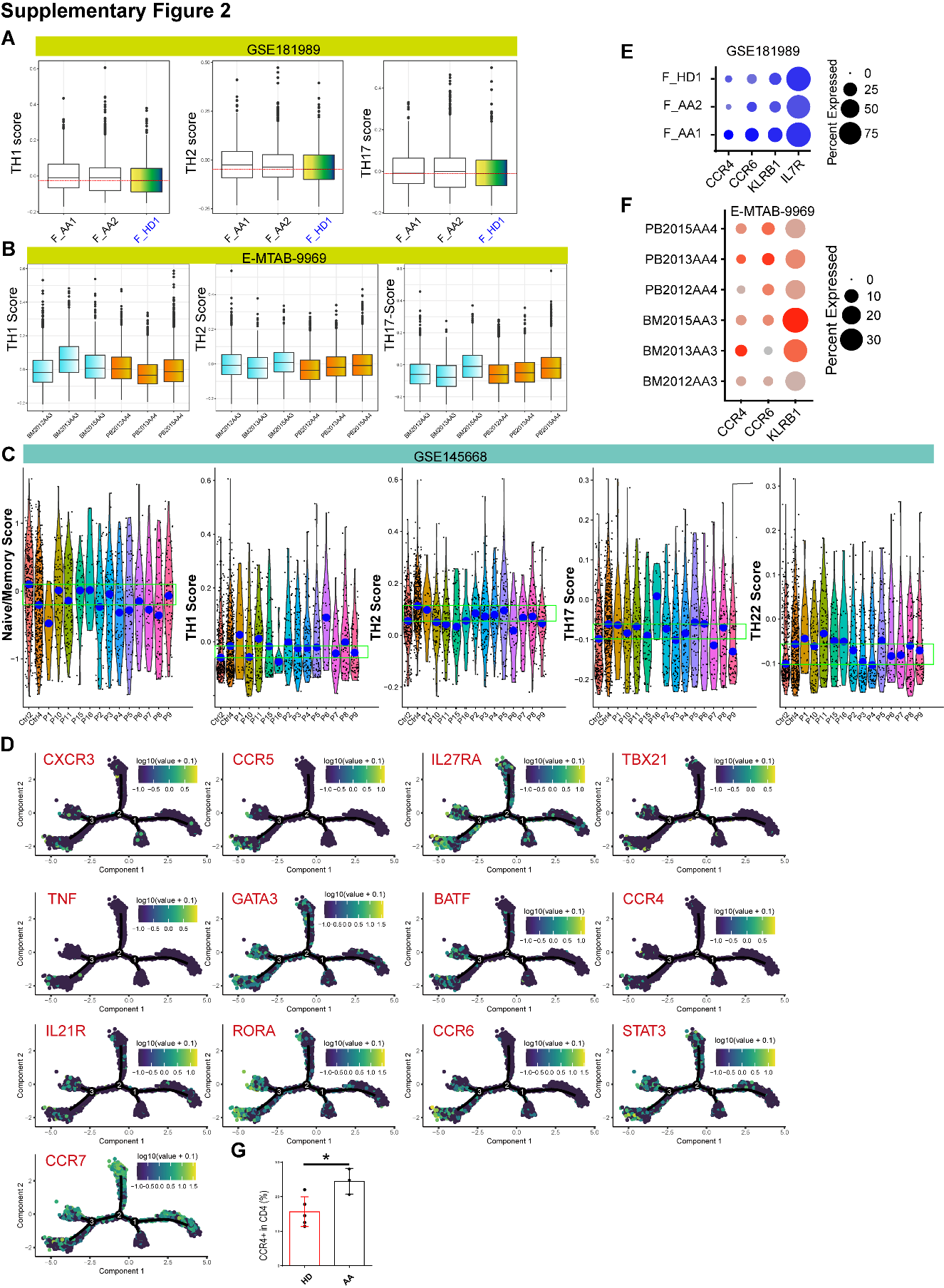


**Fig. S2.** Dysregulated naïve/memory CD4^+^ T cell homeostasis increased the differentiation of TH1, TH2, and TH17. **(A).** TH1 score, TH2 score, and TH17 score in BM CD4^+^ T cells of AA patients and HDs (GSE181989). **(B).** TH1 score, TH2 score, and TH17 score in BM or PB CD4^+^ T cells of AA patients and healthy donors (E-MTAB-9969). **(C).** TH1 score, TH2 score, TH17 score and TH22 (*CCL7*, *CCL15*, *FGF1*, *FGF5*, *FGF12*, *FGF13*, *IL13*, *IL22*, *TGFB2*, *CCL23*, *AHR*, *STAT3*, *TNFRSF1A*, *TGFBR2*, *IL6R*, *CCR4*, *CCR6*, and *CCR10*) in BM or PB CD4^+^ T cells of AA patients and HDs (GSE145668). **(D)**. Expression maps showing log-normalized expression of functional genes (*CXCR3*, *CCR5*, *IL27RA*, *TBX21*, *TNF*, *GATA3*, *BATF*, *CCR4*, *IL21R*, *RORA*, *CCR6*, *STAT3*, and *CCR7*) in the differentiation of Naïve CD4^+^ T to TH1/TH2/TH17 cells. **(E).** The dot plot showed the expression levels of *CCR4*, *CCR6*, *KLRB1*, and *IL7R* in CD4^+^ T cells of AA patients and HD (GSE181989). **(F).** The dot plot showed the expression levels of *CCR4*, *CCR6*, and *KLRB1* in CD4^+^ T cells of AA patients and HD (E-MTAB-9969). **(G).** Percentage of CCR4^+^ cells in PB CD4^+^ T cells of AA patients (n=5) and HD (n=3).


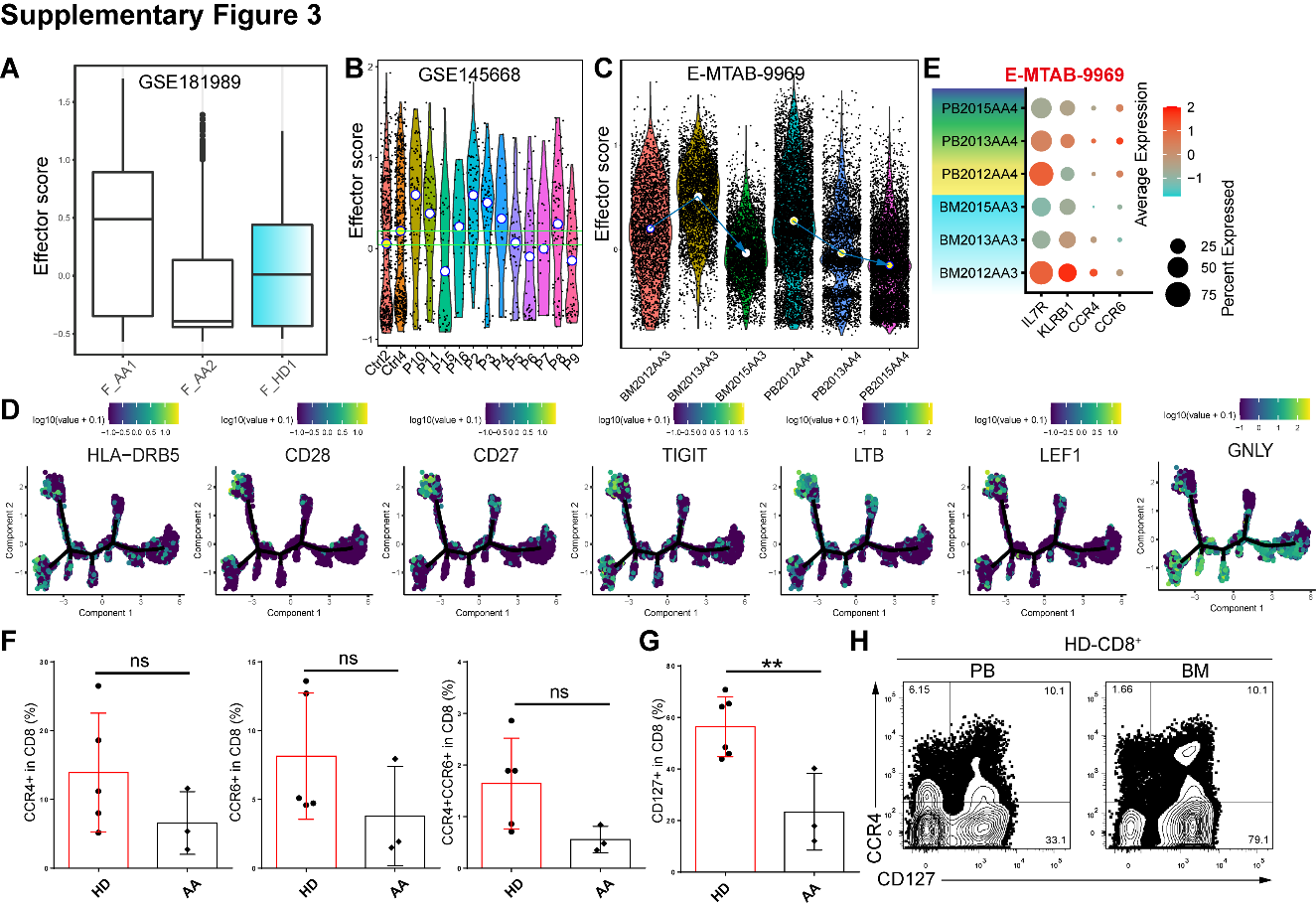


**Fig. S3.** Dysregulated naïve/memory CD8^+^ T cell homeostasis. **(A).** Effector score in BM CD8^+^ T cells of patients with AA and HD (GSE181989). **(B).** Effector score in BM CD8^+^ T cells of patients with AA and HDs (GSE145668). **(C).** Effector score in PB or BM CD8^+^ T cells of patients with AA and HD were monitored during the IST treatment (E-MTAB-9969). **(C).** Exhaustion score in BM CD8^+^ T cells of patients with AA and HD. . **(D)**. Expression maps showing log-normalized expression of functional genes (*HLA-DRB5*, *CD28*, *CD27*, *TIGIT*, *LTB*, *LEF1*, and *GNLY*) in the differentiation of Naïve CD8^+^ T to effector cells. Data were shown as log-normalized expressions. Yellow indicates high expression, and dark blue indicates low expression. **(E)** The dot plot showed the expression levels of marker genes in PB or BM CD8^+^ T cells of AA patients during the IST treatment. Percentage of CCR4^+^ cells, CCR6^+^ cells, CCR4^+^CCR6^+^ cells (AA, n=3; HD, n=5) **(F)**, and CD127^+^ cells (AA, n=3; HD, n=6) **(G)** in PB CD4^+^ T cells of patients with AA and HD. **(H).** Representative flow cytometry dot plots of CD8^+^ cytotoxic cells expressing CCR4 and CD127 in PBMC and BMMC of healthy donors.

**
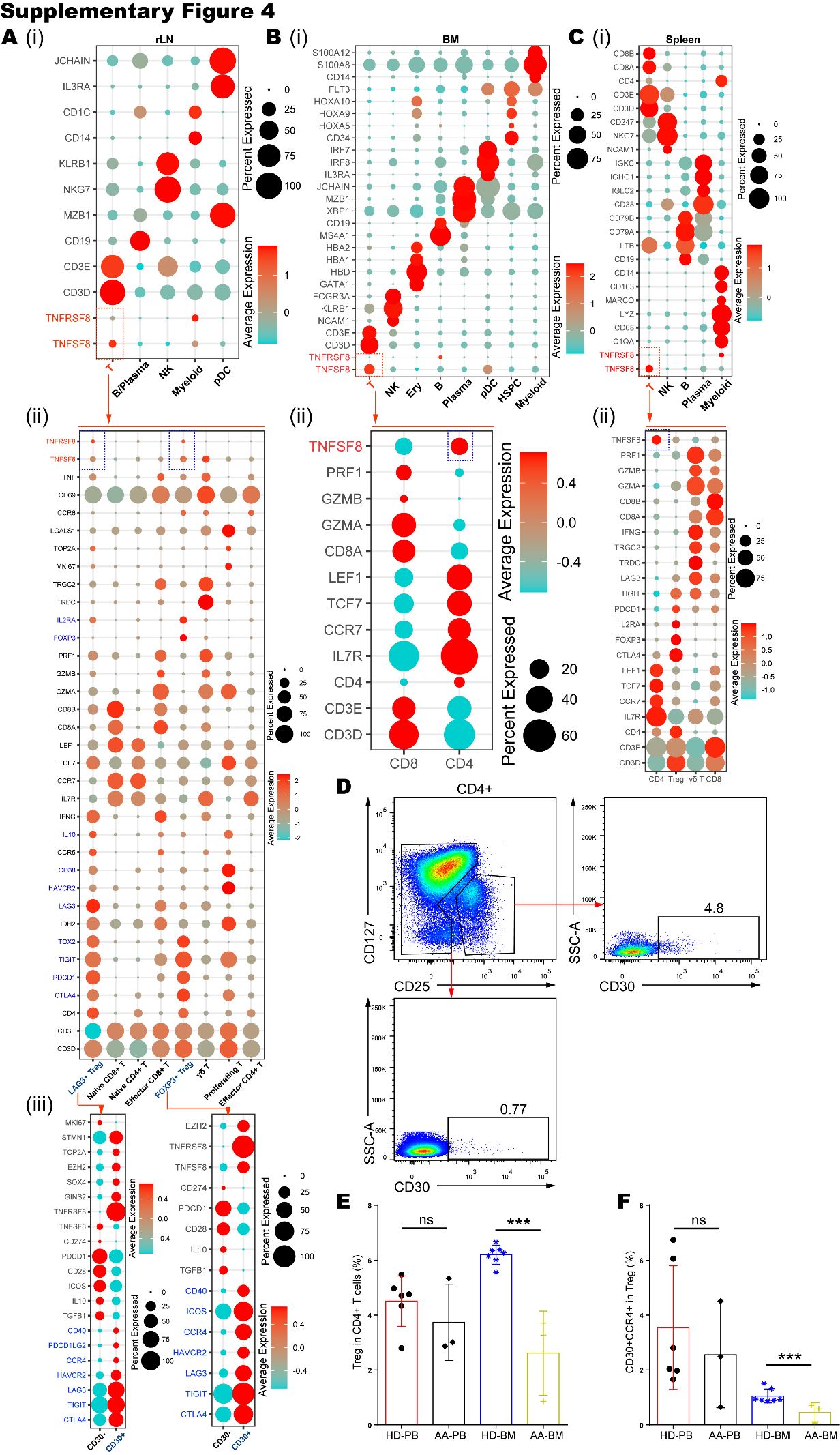
**

**Fig. S4.** Dot plot showing *TNFSF8*, *TNFRSF8*, and known markers distinguishing different subsets in reactive lymphoid nodes (rLN) (The scRNA-seq datasets were downloaded from <https://www.zmbh.uni-heidelberg.de/Anders/scLN-index.html>) **(A)**, in bone marrow (BM) (GSE116256) **(B)**, and spleen (GSE159929) **(C). (D).** Representative flow cytometry dot plots of different CD4^+^ T subsets expressing CD30 in PBMC of healthy donors. **(E).** Percentage of Treg cells in CD4^+^ T cells of PBMC and BMMC from patients with AA (PB, n=3; BM, n=3) and HDs (PB, n=6; BM, n=7). **(F).** Percentage of CD30^+^CCR4^+^ cells in Treg cells of PBMC and BMMC of patients with AA and HDs.


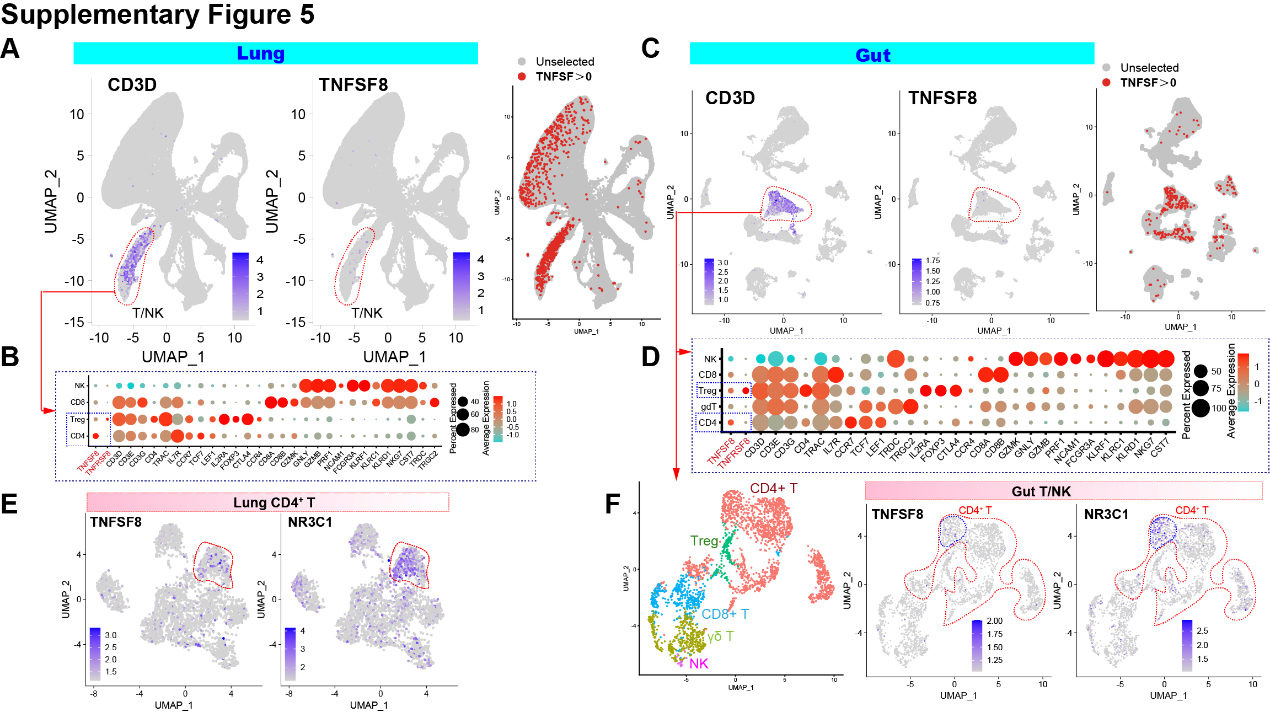


**Fig. S5.** UMAP plots revealing the *CD3D* and *TNFSF8* expression in lung-derived cells **(A)** and intestine-derived cells **(C)**. Dot plots of *TNFSF8*, *TNFRSF8*, and typical markers on different T and NK subsets from lung **(B)** and gut **(D)**. UMAP plots showing the expression of *TNFSF8* and *NR3C1* in lung-derived CD4+ T cells **(E)** and intestinal T/NK cells **(F)**.


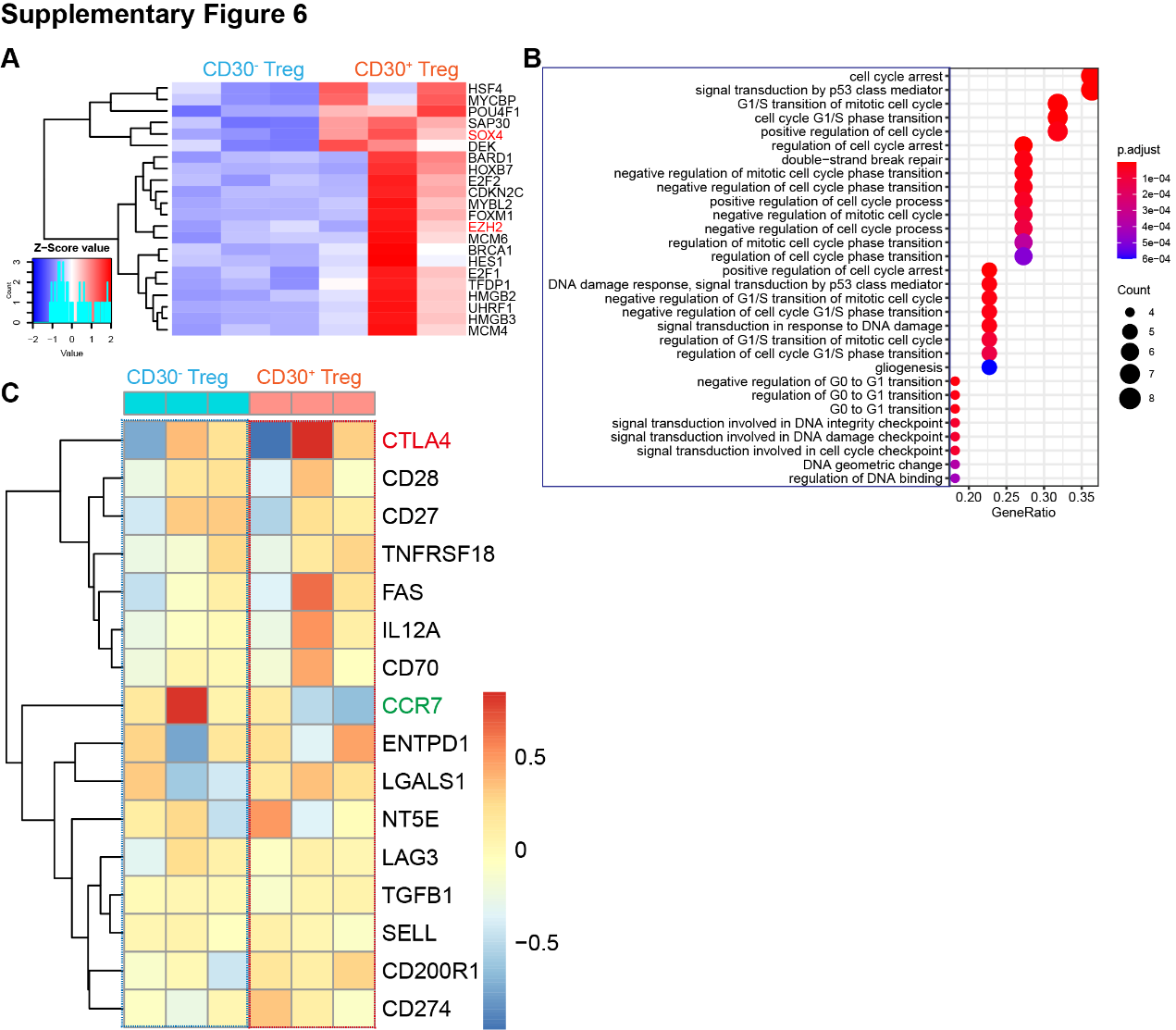


**Fig. S6. (A).** Heatmap showing upregulated differential transcription factors of CD30^+^ Treg cells. **(B).** Representative GO terms enriched in upregulated transcription factors of CD30^+^ Treg cells. **(C).** Heatmap showing immunosuppressive phenotypical genes.

**Table S1. Clinical information of each sample.**

| **No.** | **Age (year)** | **gender** | **Disease classification** | **Interval from onset to diagnosis (m)** | **Peripheral blood count** | | | | | **Bone marrow hypoplasia** | **Cytogenetic** | **Genetic mutation** | **Treatment** |
| --- | --- | --- | --- | --- | --- | --- | --- | --- | --- | --- | --- | --- | --- |
|  |  |  |  |  | **WBC(*10e9/L)** | **ANC(*10e9/L)** | **HGB(g/L)** | **PLT(*10e9/L)** | **ARC(*10e9/L)** |  |  |  |  |
| **AA1** | **50** | **M** | **sAA** | **3** | **0.25** | **0.03** | **29.4** | **22** | **2.3** | **＜5** | **NA** | **NA** | **CyA, G-CSF** |
| **AA2** | **57** | **M** | **sAA** | **7** | **1.3** | **0.02** | **29.8** | **2** | **0.02** | **＜5** | **NA** | **NA** | **TPO-RA, G-CSF** |
| **HD1** | **27** | **F** | **--** | **--** | **5.6** | **2.58** | **128** | **315** | **NA** | **NA** | **NA** | **NA** | **--** |
| **HD2** | **27** | **F** | **--** | **--** | **6.79** | **3.52** | **139.9** | **412.3** | **NA** | **NA** | **NA** | **NA** | **--** |

Note: WBC, White Blood Cell Count; ANC, absolute neutrophil count; HGB, hemoglobin; PLT, Platelet count; ARC, Absolute Reticulocyte Count.
